# A multi-hazard map-based flooding, gully erosion, forest fires, and earthquakes in Iran

**DOI:** 10.1038/s41598-021-94266-6

**Published:** 2021-07-21

**Authors:** Soheila Pouyan, Hamid Reza Pourghasemi, Mojgan Bordbar, Soroor Rahmanian, John J. Clague

**Affiliations:** 1 Department of Natural Resources and Environmental Engineering, College of Agriculture, Shiraz University, Shiraz, 71441-65186 Iran; 2 Department of GIS/RS, Faculty of Natural Resources and Environment, Science and Research Branch, Islamic Azad University, Tehran, Iran; 3 Quantitative Plant Ecology and Biodiversity Research Lab, Department of Biology, Faculty of Science, Ferdowsi University of Mashhad, Mashhad, 9177948974 Iran; 4 Department of Earth Sciences, Simon Fraser University, 8888 University Drive, Burnaby, BC V5A 1S6 Canada

**Keywords:** Environmental sciences, Natural hazards

## Abstract

We used three state-of-the-art machine learning techniques (boosted regression tree, random forest, and support vector machine) to produce a multi-hazard (MHR) map illustrating areas susceptible to flooding, gully erosion, forest fires, and earthquakes in Kohgiluyeh and Boyer-Ahmad Province, Iran. The earthquake hazard map was derived from a probabilistic seismic hazard analysis. The mean decrease Gini (MDG) method was implemented to determine the relative importance of effective factors on the spatial occurrence of each of the four hazards. Area under the curve (AUC) plots, based on a validation dataset, were created for the maps generated using the three algorithms to compare the results. The random forest model had the highest predictive accuracy, with AUC values of 0.994, 0.982, and 0.885 for gully erosion, flooding, and forest fires, respectively. Approximately 41%, 40%, 28%, and 3% of the study area are at risk of forest fires, earthquakes, floods, and gully erosion, respectively.

## Introduction

Hazard can be defined as a source of potential harm, a threat to natural environments, and human health. The causes of natural hazards may be geological (e.g., earthquakes, tsunamis, landslides, volcanic eruptions, etc.) and climatic (e.g., floods, windstorms, droughts, and wildfires)^1^. Many parts of the world are at risk from one or more natural hazards, and although many studies have focused on single hazards, there is a need for integrated assessments of multi-hazards for more efficient land management^2^. The concept of multi-hazards was introduced by the United Nations Environment Programme through its policies on sustainable development and its call for "comprehensive investigation of multi-hazards" to plan and manage residential areas prone to natural disasters^3^. Damage from natural disasters is increasing worldwide, providing an impetus to hazard researchers to develop new tools to reduce economic losses and injuries from future disasters. These tools include multi-hazard maps created using machine learning tools that show the spatial distribution of the full spectrum of hazards in a region^2^. Examples of areas where such multi-hazard evaluations have been performed include Greece (flooding depth, lateral erosion, earthquakes, and landslides)^4^, the United States (weather and climate hazards)^5^, Sikkim State, India (landslide and earthquake)^6^, the Adriatic Sea (smothering and sealing, abrasion and extraction, underwater noise, sea surface temperature variation, and sea surface salinity variation)^7^, and Chile (weather-related hazards, including coastal flooding, fluvial flooding, water scarcity, heat stress, and wildfire)^8^. The interaction of these multi-hazards could be useful for further research in this field. Previous studies have shown that landslides and flash floods are natural hazards that are frequently triggered simultaneously due to heavy or prolonged rainfall on steep mountains^9^. In other words, heavy rainfall causes flash floods that can lead to soil erosion and landslide events^10^. For instance, in April 2019, heavy rainfall had a significant effect on the whole of Iran, which caused far-reaching flooding and landslides^11^. Furthermore, landslides lead to seismic derangement. In addition, seismic derangement may cause landslides, leading to many victims around the world. Slides can cause catastrophic flooding, especially when landslide dams are broken, and flooding can cause slides^12^. Temporary soil flooding at different scales can significantly affect soil degradation. Floods on slopes in ground flow, sheet flow, return flow, groundwater ridge, etc., are connected to soil erosion and landslide occurrence. Floodwater combined with saturated status may destroy soil structure and soil organisms (https://www.recare-hub.eu/soil-threats/floods-and-landslides). Moreover, forest fires can significantly alter vegetation, increase soil erosion, and even lead to desertification^13^. Admittedly, forest fires significantly impact physical, chemical, mineralogical, and biological soil features, which trigger soil erosion. Severe bushfires (i.e., wildfires) harm soil properties^14^. On the other hand, gully erosion influences soil structure, quality, and soil properties^15^.

Much of Iran is at high risk of earthquakes, floods, forest fires, and gully erosion^16–18^. Forest fires, for example, are a major cause of ecosystem damage and large economic losses^19, 20^. The forests of Kohgiluyeh and Boyer-Ahmad Province in southwestern Iran are particularly vulnerable to forest fires, providing a motivation for the choice of this area for our study. Maps that show areas at high risk from wildfires help managers and planners to deal with this problem^21, 22^.

Flooding is another serious hazard in Kohgiluyeh and Boyer-Ahmad Province and has been exacerbated in recent decades by deforestation, land use changes, poor watershed management, and climate change^23^. Floods have adversely affected more people than any other natural disaster type in the twenty-first century, including 127 events of differentiated natural disasters in 2018^24^. One such flood caused widespread damage in Iran in March and April 2019. Recurrent floods in Kohgiluyeh and Boyer-Ahmad Province inundated riverside areas, leading to soil erosion, damaged or destroyed earth and concrete dams, other watershed structures, and bridges. Identifying areas at risk^25^ and preparing flood hazard maps are important activities in any proactive response to this hazard^26–29^.

Gully erosion is an important cause of soil loss and land degradation in arid and semi-arid regions in Iran and elsewhere world^30, 31^. Maps showing where this problem exists or might develop in the future are effective tools for decision makers concerned with sustainable development. In many instances, gully erosion maps have been prepared individually based on datasets in GIS software^5, 32, 33^. In the present study, gully erosion is considered an element of the multi-hazard spectrum hazards in the Kohgiluyeh and Boyer-Ahmad Province.

The high Zagros Mountains throughout the north, east, and southeast of Kohgiluyeh and Boyer-Ahmad Province is located, experience frequent earthquakes. Although it is not possible to accurately predict future earthquakes in the province, it is possible to determine the likely locations that will suffer damaging ground motions.

Our multi-hazard study in Kohgiluyeh and Boyer-Ahmad Province involved (1) identifying and prioritizing the factors affecting the occurrence of each natural hazard in the region; (2) preparing maps of flood, gully erosion, forest fires, and earthquake hazards separately using three machine learning algorithms (boosted regression tree, support vector machine, and random forest); (3) comparing the results of the three algorithms with the ROC curve to select the best regional hazard model; and (4) preparing a multi-hazard map of the province. Our research contributes to the development and assessment of machine learning methods for mapping natural hazard zones. To the authors’ knowledge, no work in the literature related to the multi-hazard spatial modeling of floods, gully erosion, forest fires, and earthquakes exists to date. Moreover, for the first time, this work was carried out mentioned hazards mapping in the Kohgiluyeh and Boyer-Ahmed province. In addition, based on the available data and sources, the SVM, BRT, and RF algorithms were used to investigate the hazards in the study area.

## Results

### Priority of effective factors using random forest and MDG

**Figure 1:**
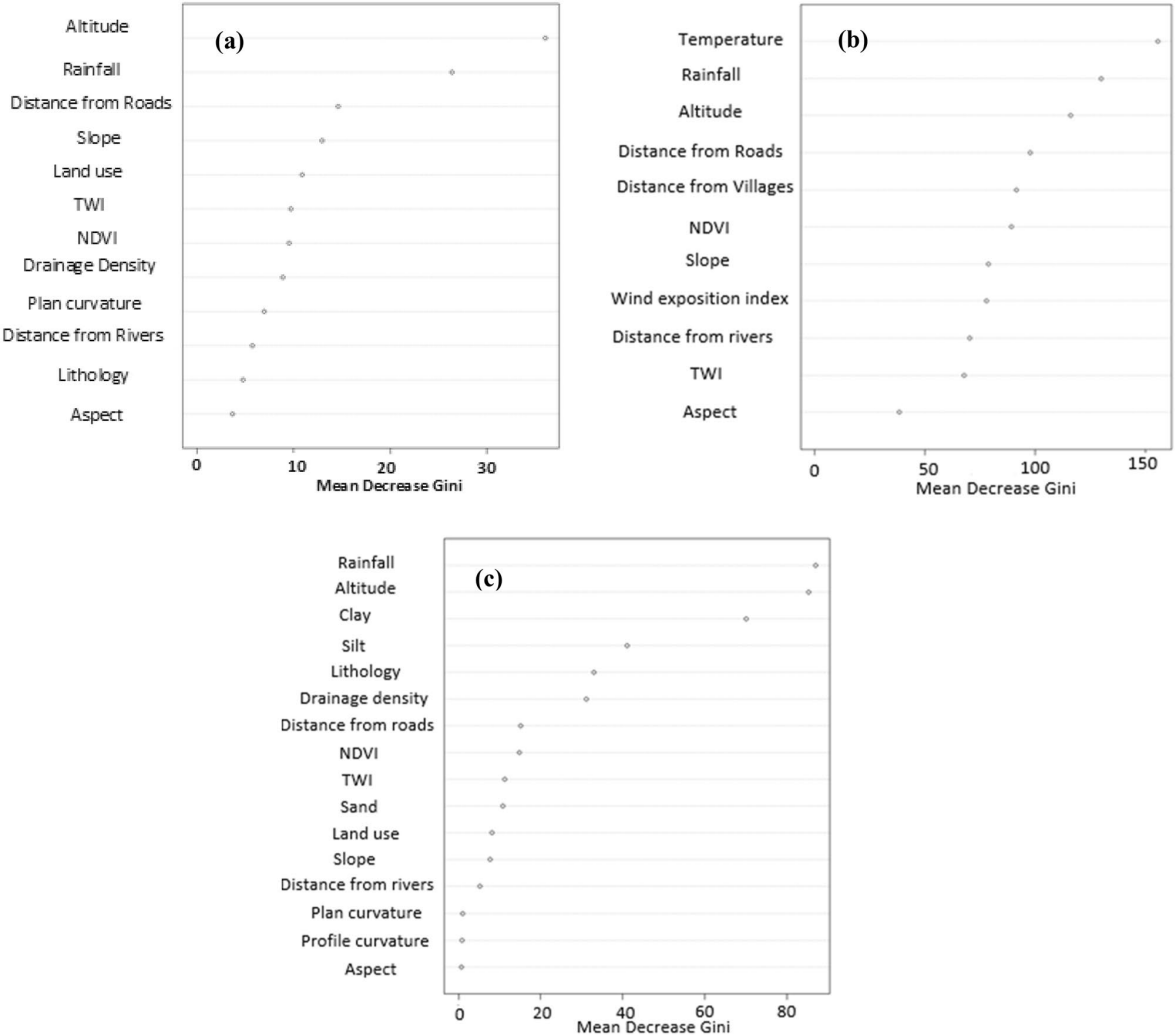
Priority of the effective factors for each hazard.

Figure 1 shows the results of the prioritization of factors for the three hazards using the RF technique. The most important factor is elevation, followed by decreasing order of importance of flooding by mean annual rainfall, distance from roads, slope, land use, TWI, NDVI, drainage density, plan curvature, distance from rivers, lithology, and aspect (Fig. 1a).

Based on the MDG analysis, temperature is the most important factor for forest fire hazards, followed by mean annual rainfall, elevation, distance from roads, distance from villages, NDVI, slope, WEI, distance from rivers, TWI, and aspect (Fig. 1b). Mean annual rainfall is the most important factor for gully erosion, followed by elevation, clay, silt, lithology, drainage density, distance from roads, NDVI, TWI, sand, land use, slope, distance from rivers, plan curvature, profile curvature, and aspect (Fig. 1c).

### Natural hazard susceptibility maps

In natural hazard studies, susceptibility is associated with the spatial features of hazards. It is defined as the tendency of a region to experience the effects of a given hazardous process (i.e., earthquakes, floods, forest fires, erosion, etc.) without considering the moment of event, fatality, and economic losses^34^. The RF, BRT, and SVM algorithms were applied to train the data, and susceptibility maps were prepared for flood, forest fire, and gully erosion hazards. Flood hazard (FH) maps were created by applying the RF (FHRF), SVM (FHSVM), and BRT (FHBRT) algorithms to the training dataset. Similarly, gully erosion hazard (GEH) maps were generated from the training dataset using the same three algorithms (maps GEHRF, GEHSVM, and GEHBRT), as were forest fire hazard (FFH) maps (FFHRF, FFHSVM, and FFHBRT).

**Figure 2:**
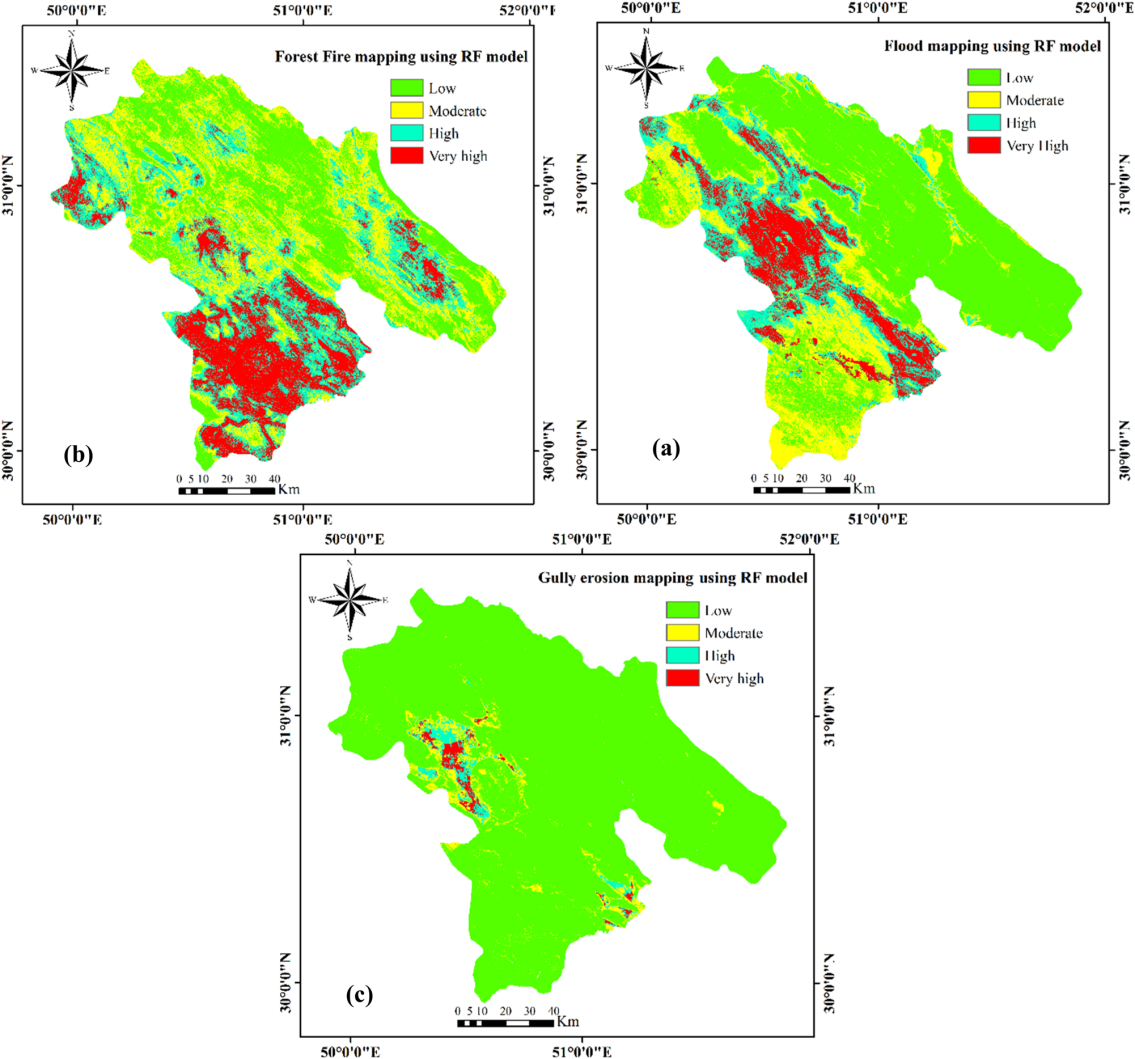
Hazard susceptibility maps of the study area produced using the RF model.

Natural hazard maps prepared using the RF model (the best performance of the three models) are shown in Fig. 2. In the case of the FHRF map (Fig. 2a), approximately 50.1%, 22.1%, 15.7%, and 12.1% of the study area had low, moderate, high, and very high susceptibility to flooding, respectively. The FFHRF map (Fig. 2b) shows that approximately 17.4% and 24.0% of the study area are, respectively, in the high and very high forest fire susceptibility classes, and 30.3% and 28.3% of the area had moderate and low susceptibility, respectively. Finally, the GEHRF map (Fig. 2c) shows that 1.1% and 2.1% of the area has very high and high susceptibility to gully erosion, respectively; the moderate and low susceptibility classes cover 4.4% and 92.4% of the province, respectively.

### Peak ground acceleration map

**Figure 3:**
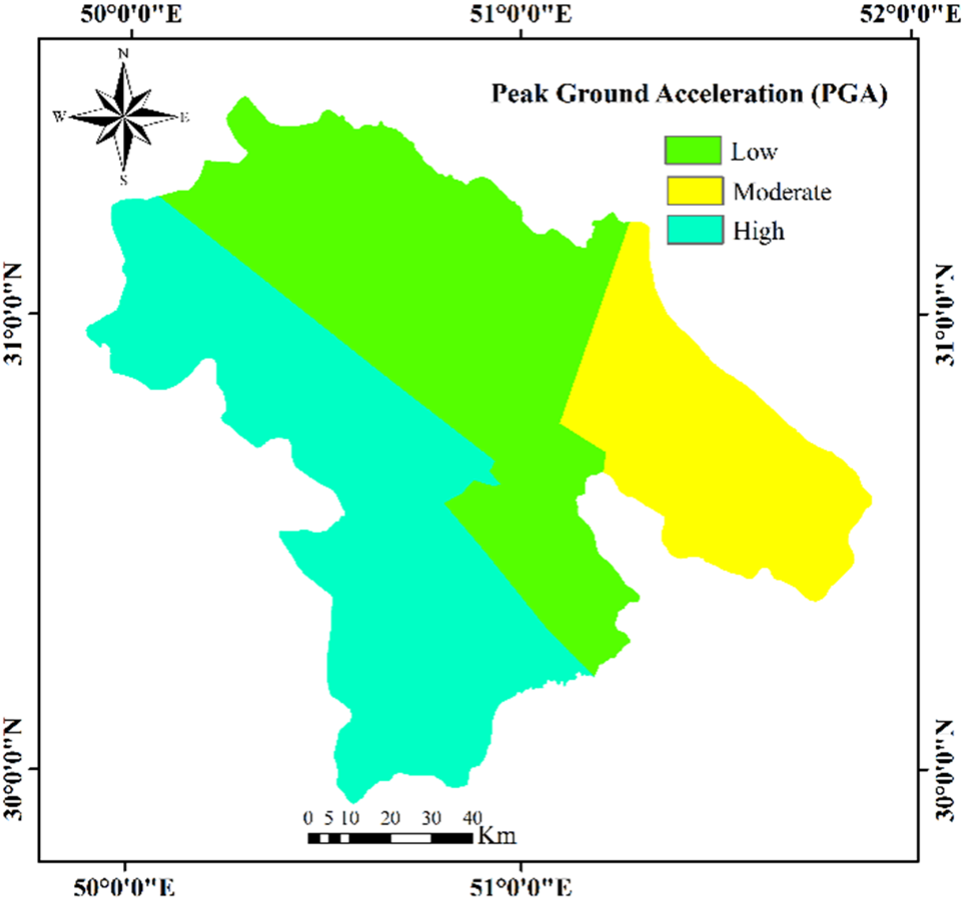
PGA map.

Figure 3 shows the PGA map of the study area. The PGA values were divided into three classes: low, moderate, and high. The PGA map shows that approximately 40%, 20.0%, and 39.6% of the area were in the low, moderate, and high classes, respectively.

### Validation and comparison of hazard maps using ROC

**Figure 4:**
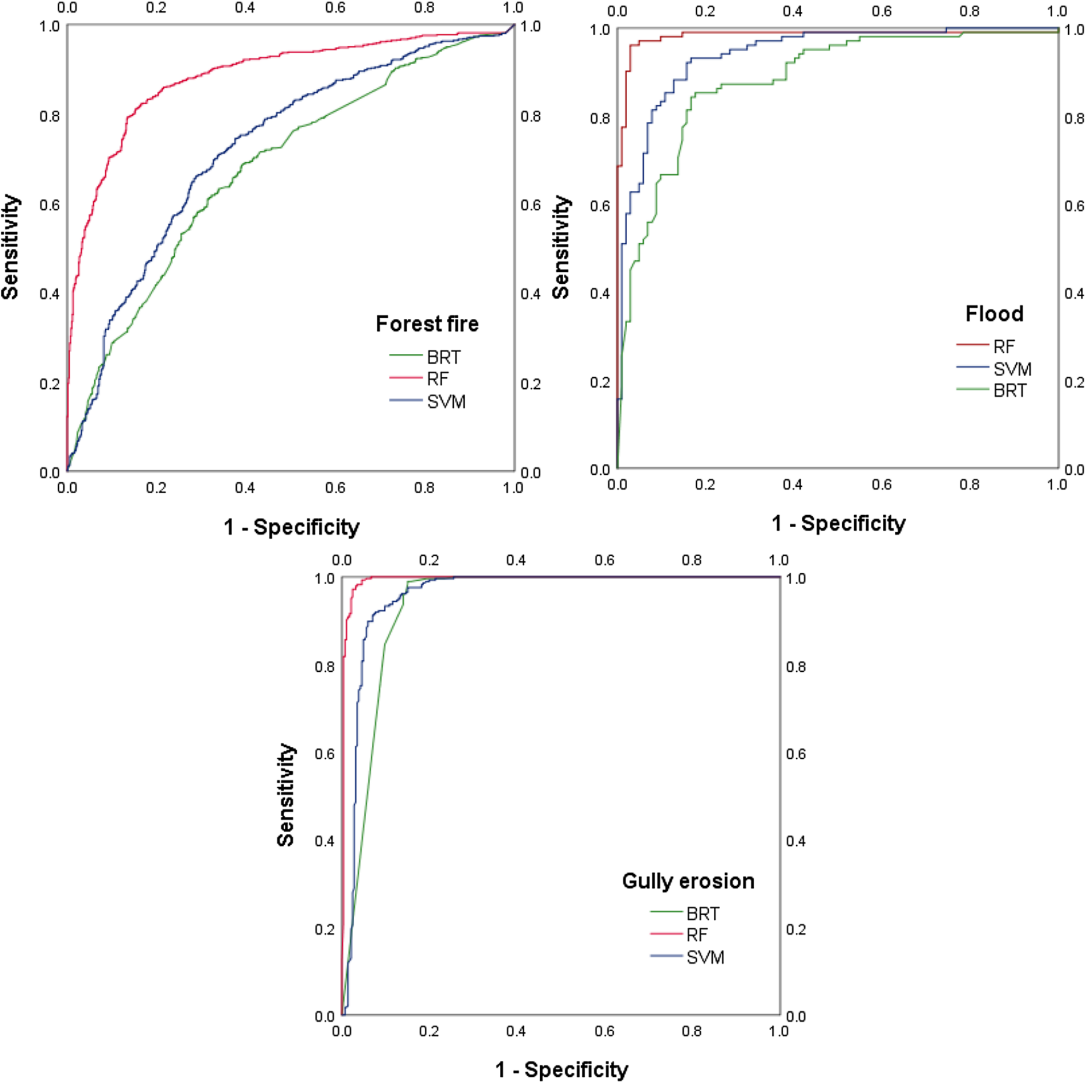
Validation of hazard maps using the ROC curve.

We validated the hazard maps using ROC (Table 1). In the case of forest fire hazard, the random forest model performed best (AUC = 0.885), followed by the support vector machine (AUC = 0.727) and boosted regression tree (AUC = 0.680) models. For gully erosion, the AUC values for the random forest, support vector machine, and boosted regression tree maps are 0.994, 0.959, and 0.938, respectively. The AUC values for the flood hazard maps made using the random forest, support vector machine, and boosted regression tree algorithms are 0.982, 0.940, and 0.883, respectively (Table 1) (Fig. 4).

**Table 1:** AUC values of hazard risk maps.

Models	Forest fire	Flood area	Gully erosion
BRT	0.68	0.883	0.938
RF	0.885	0.982	0.994
SVM	0.727	0.94	0.959

### Multi-hazard map

The RF model was chosen to produce a multi-hazard map (MHR) of the study area (Fig. 4). We combined maps of the four hazards (flood, forest fire, gully erosion, and earthquake) in the ArcGIS 10.8 environment (https://www.esri.com) using the following equation:

1 $${\text{MHR}} = {\text{FFHRF}} + {\text{FHRF}} + {\text{GEHRF}} + {\text{PGA}}$$

**Figure 5:**
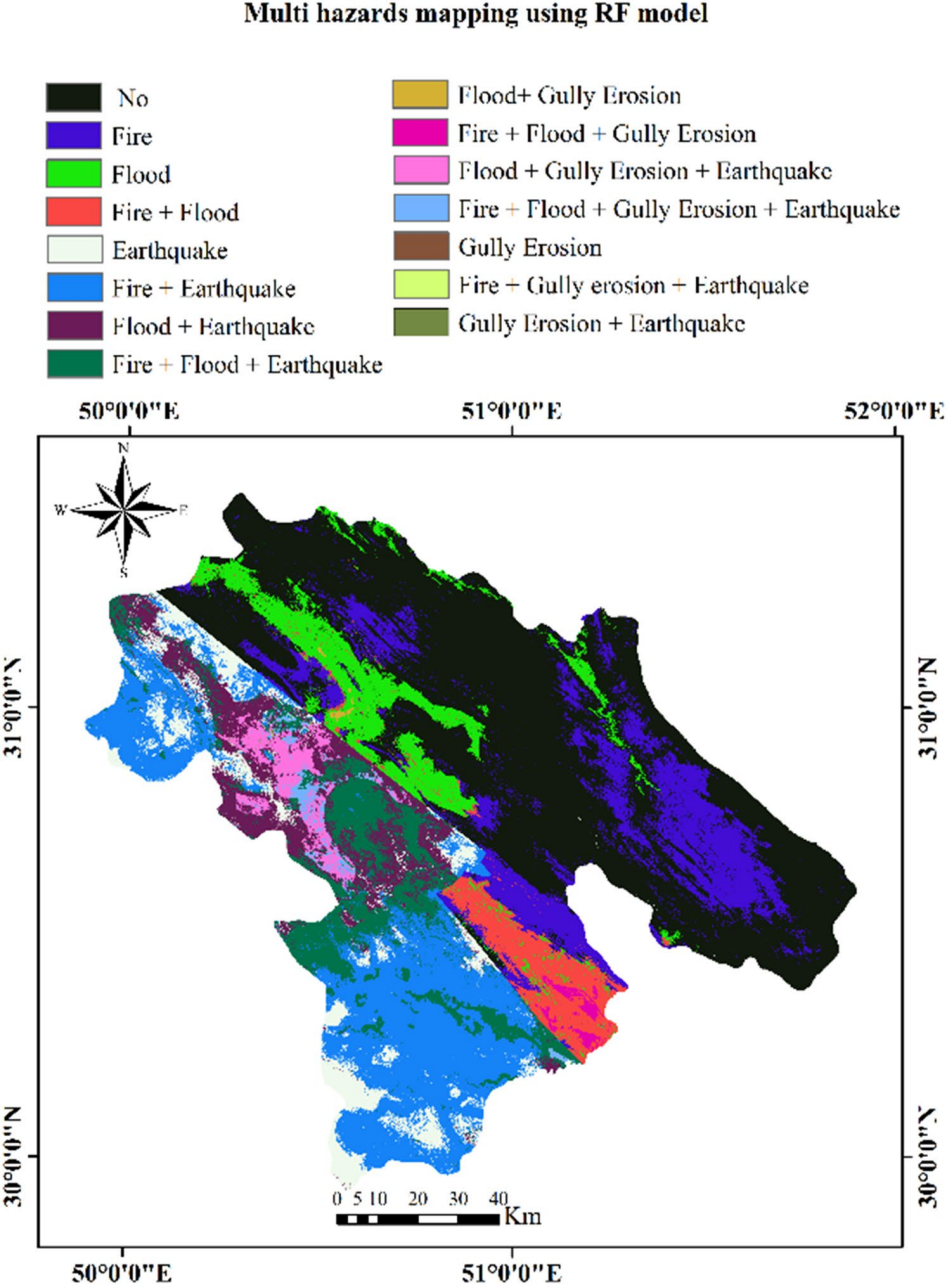
Multi-hazard map.

**Figure 6:**
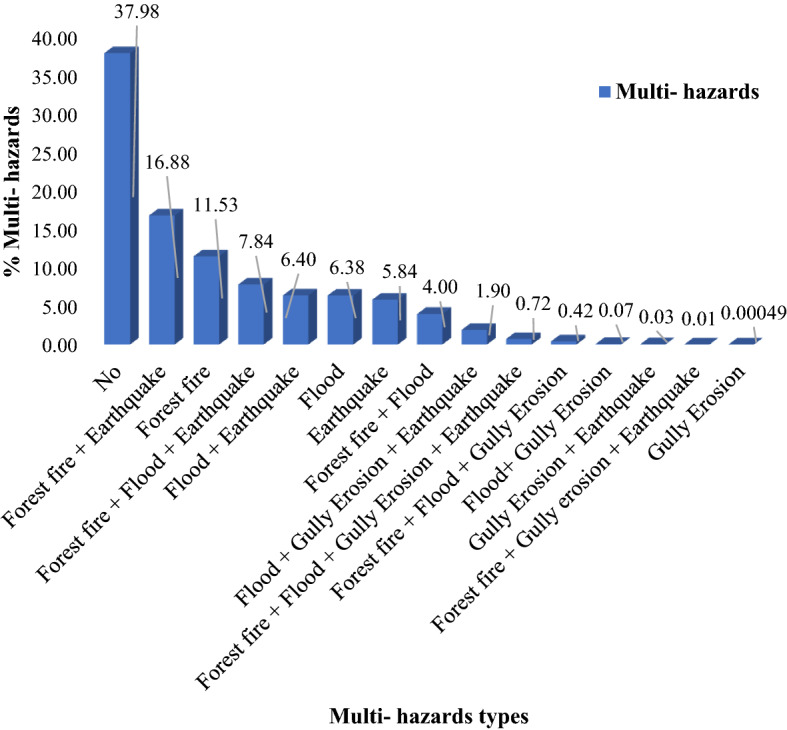
Percentages of areas of hazard classes.

**Figure 7:**
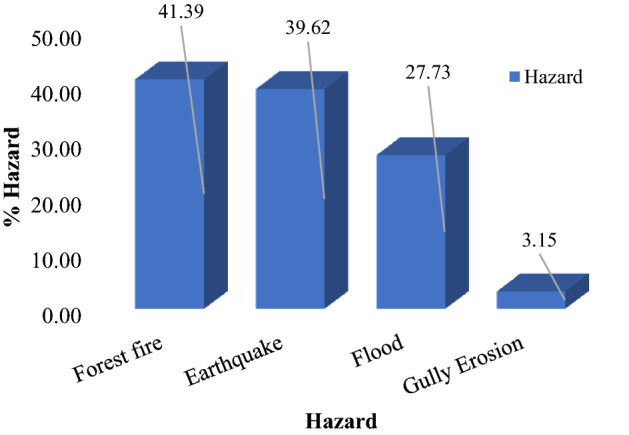
Percentage area of the four hazards.

The multi-hazard map included 15 susceptibility classes (Fig. 5). Approximately 38% of the Kohgiluyeh and Boyer-Ahmad Province is safe from all-natural hazards, whereas 0.7% of the region is susceptible to all four natural hazards. Percentages of the study area with individual or combinations of hazards are shown in Figs. 6 and 7.

## Discussion

Many researchers have studied hazards individually^35–39^. Although such studies have been important and useful, considering multiple hazards and their interactions, their interdependencies, and possible cascading effects can be more informative and useful for reducing disaster losses, as well as for efficient land and ocean management^4, 6, 40^.

The motivation for our study was to provide (1) a comprehensive and integrated analysis of the full spectrum of damaging natural hazards in Kohgiluyeh and Boyer-Ahmad Province, which is a risk-prone area in Iran, and (2) a multi-hazard map useful to land-use and emergency response managers. Here, we discuss the factors that drive each hazard and identify areas within the province that are susceptible to each phenomenon. We also discuss the merits of the machine-learning algorithms used in this study. The benefits of machine learning models include the use of dichotomous dependent variables as probabilities, the ability to use different types of independent variables (i.e., binary, sequential, and continuous), and there is no need for normal distribution^41^. Machine learning methods can solve uncertainty factors related to the dataset modeling process. Different learning machine algorithms can also be used to solve uncertainties related to the accuracy of the models. Another source of uncertainty is the limitation of the training model, in which ML techniques such as RF do not face these issues. This algorithm uses the error rate and an indicator outside the bag indicator. One of the benefits accrued from machine learning techniques compared to traditional methods (i.e., bivariate and multivariate statistical methods) is that ML algorithms can deal with noise related to the dataset and the uncertainty of the dataset. Moreover, limited measurement errors are accurate^42^. However, the disadvantage of machine learning methods is their vulnerability to overfitting data, which produces unstable regression coefficients^43^. Therefore, it is necessary to use different techniques to improve the accuracy of prediction results.

Each of these algorithms has advantages, but the results of the AUC-based assessment showed that the RF algorithm has stronger predictive power than the BRT and SVM models. Other researchers have shown that (1) the RF algorithm performs better than conventional methods^44^, (2) is a powerful supervised learning method for investigating problems in the real world^45^, (3) is simple and fast, (4) does not require statistical hypotheses, and (5) is a reliable predictor^46, 47^. It has been widely used in a range of environmental studies^44, 48–52^.

The FFHRF map shows that the southern, western, and eastern parts of the Kohgiluyeh and Boyer-Ahmad Province are most susceptible to forest fires, and the northern and central areas have low to moderate wildfire susceptibility. The FHRF map shows flood susceptibility to be high to very high in the western and southeastern parts of the province and low to moderate s in the north and east. According to the GEHRF map, high to very high gully erosion susceptibility is restricted to a small area in the western part of the province. Finally, the PGA map shows that the southern and western parts of the province are highly susceptible to damaging earthquakes, whereas this hazard is low elsewhere.

In this study, we evaluated the relative importance of factors affecting flood, forest fire, and gully erosion using the random forest method. This method reliably determines the relative importance of controlling factors in hazard susceptibility applications^53^. Using the MDG method, we found that the annual temperature is the most important factor in predicting forest fire susceptibility, whereas elevation and mean annual precipitation are most important in the case of flood and gully erosion hazards. Aspect proved to be the least important factor for all three hazards. There is no specific aspect that favors forest fires; it occurs on all forested slopes in the study area. However, forest fires are common near residential areas and roads, where most fires are initiated. Elevation is also important because it has a strong effect on rainfall and temperature. Our map of areas susceptible to forest fires may be useful to forest managers and emergency responders who must plan and implement necessary measures to protect and preserve the remaining forest in high-risk areas.

Researchers used the RF method in a forestry study in China and, like us, concluded that vegetation type, slope, and aspect are less important than proximity to towns, temperature, and precipitation^54^. Another research group studied flood susceptibility along the Pearl River in China using the DMG method and concluded that the most important factors for related flooding are maximum three-day precipitation, runoff depth, typhoon frequency, elevation, and TWI^55^. In contrast, another group found that elevation is the most important flood risk factor in their study area^56^, given that flooding was greatest along trunk rivers at low elevation.

Mean annual rainfall is one of the most effective factors in the formation and spread of gullies. This metric is positively related to the volume and kinetic energy of surface runoff, which are the main factors involved in gully erosion^57^. Lithology is an important factor in this process. One research group working in Ilam Province, Iran, for example, found that the amount of clay in soil has a high positive correlation with gully erosion^58^.

In our analysis of earthquake hazards, we learned that magnitude and ground acceleration are the two most important factors for estimating seismic risk. This finding is consistent with the results of other studies that have performed similar studies^40^.

## Conclusion

We used machine learning algorithms to map areas susceptible to multiple hazards in a part of Iran with a high risk of natural hazards. Identifying these areas is an important step in making sound management decisions to reduce damage and injury from natural hazards in the future. The final product of our work is a multi-hazard map created by combining four important natural hazards (i.e., flooding, forest fires, earthquakes, and gully erosion). Factors affecting each hazard were identified and prioritized, and maps of each hazard were created using the RF, BRT, and SVM algorithms. We determined the validity of the results using ROC plots and found that the RF model had the highest AUC value and thus accuracy. We then produced a multi-hazard map for the study area by combining the maps of the four hazards. This final map shows that approximately 38% of the province is safe from all four hazards. Areas where all four hazards are a concern are restricted to the southern part of the province. Of the four hazards, forest fires affected the largest percentage of the study area and gully erosion was the least. In line with the goals of sustainable development, the results of this research can be used by managers, planners, and other stakeholders as a decision-making tool to reduce future damage from natural hazards. The critical infrastructure in Kohgiluyeh and Boyer-Ahmad Province should be examined with the aim of minimizing future losses from forest fires, floods, earthquakes, and gully erosion.

## Methodology

### Study area

**Figure 8:**
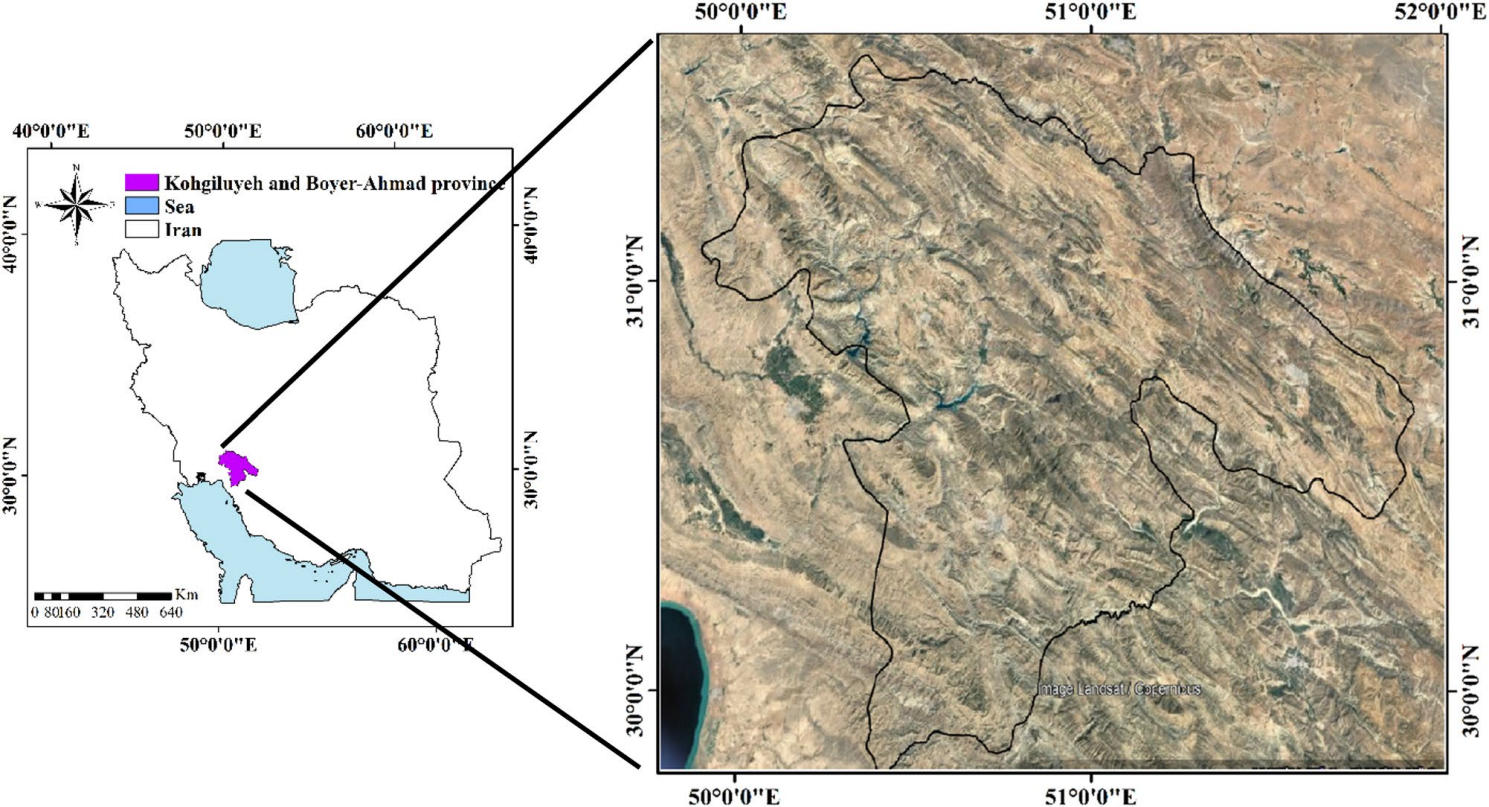
Location of the Kohgiluyeh and Boyer-Ahmad Province in Iran.

Kohgiluyeh and Boyer-Ahmad Province is a 15,500 km^2^ area of a high mountainous region. Its borders share with Fars, Khuzestan, Isfahan, and Bushehr provinces within the Chaharmahal-Bakhtiari province, and the Zagros Mountains in southwest Iran (Fig. 8). It is located between 49° 53′ 00″ and 51° 53′ 00″ E longitudes and 29° 56′ 00″ and 31° 27′ 00″ N latitudes, and altitude from 109 to 4294 m, and has an average annual rainfall of 550.7 mm and the average temperature is between 15 and 26 °C. Plains constitute approximately 20% of province. The highest point in the province is Dena Mountain (4294 m above sea level); the lowest point is in the southwest, in the city of Gachsaran, (109 m above sea level. The difference in elevation between the more elevated northeastern part of the province and the southern and southwestern parts results in two different climatic regimes. The former region is cooler and drier than the latter^59^.

### Preparation of inventory maps

Based on experience and expert opinion, we consider the four main hazards in the study area to be forest fires, flooding, earthquakes, and gully erosion. In this study, we identified the locations of these four hazard types using Google Earth images and a field survey supported by a global positioning system (GPS). In this study, floods were investigated in 2000. Training and test data were prepared from 2000 to 2020. In addition, data from 2019 and 2020 were extracted using Sentinel satellite imagery. However, in the article, we only mentioned the data for 2019 and 2020; therefore, more details were added. Areas affected by these events were identified using the Google Earth Engine (GEE) and high-resolution Sentinel-1 and -3 images. We identified forest fire locations for the period 2015–2020 based on the GEE images. The codes were written in the GEE environment (https://earthengine.google.com). We randomly split the flood, gully erosion, and forest fire locations into two groups: a model training dataset with 70% of the locations, and a validation dataset with 30% of the locations^60^. The earthquake map was created from the epicenters and magnitudes of historic earthquakes provided by S.A.P. Consulting Engineers Co the Kohgiluyeh and Boyer-Ahmad Deputy Governor of Planning (www.sabzandish.com).

### Effective factors for multi-hazard assessment

The first step in mapping and assessing natural hazards is to choose the appropriate control variables. Based on data availability and past experience^61–66^, we employed 19 effective factors in this study: elevation, slope, aspect, mean annual rainfall, mean annual temperature, lithology, land use, normalized difference vegetation index (NDVI), soil texture (percent clay, silt, and sand), wind exposure index, topographic wetness index (TWI), plan curvature, profile curvature, drainage density, distance from roads, distance from rivers, and distance from villages (Table 2). These factors were rasterized with a 30 × 30 m pixel size in the ArcGIS 10.8 platform and are briefly described below.

**Table 2:** The effective factors for MHA.

Effective factor	Flood	Forest fire	Gully erosion
Elevation	*	*	*
Slope	*	*	*
Aspect	*	*	*
Mean annual rainfall	*	*	*
Mean annual temperature		*	
Lithology	*		*
Land use	*		*
NDVI	*	*	*
Soil texture (clay)			*
Soil texture (silt)			*
Soil texture (sand)			*
Wind exposition index		*	
TWI	*	*	*
Plane curvature	*		*
Profile curvature			*
Drainage density	*		*
Distance from roads	*	*	*
Distance from rivers	*	*	*
Distance from villages		*	

#### Elevation

**Figure 9:**
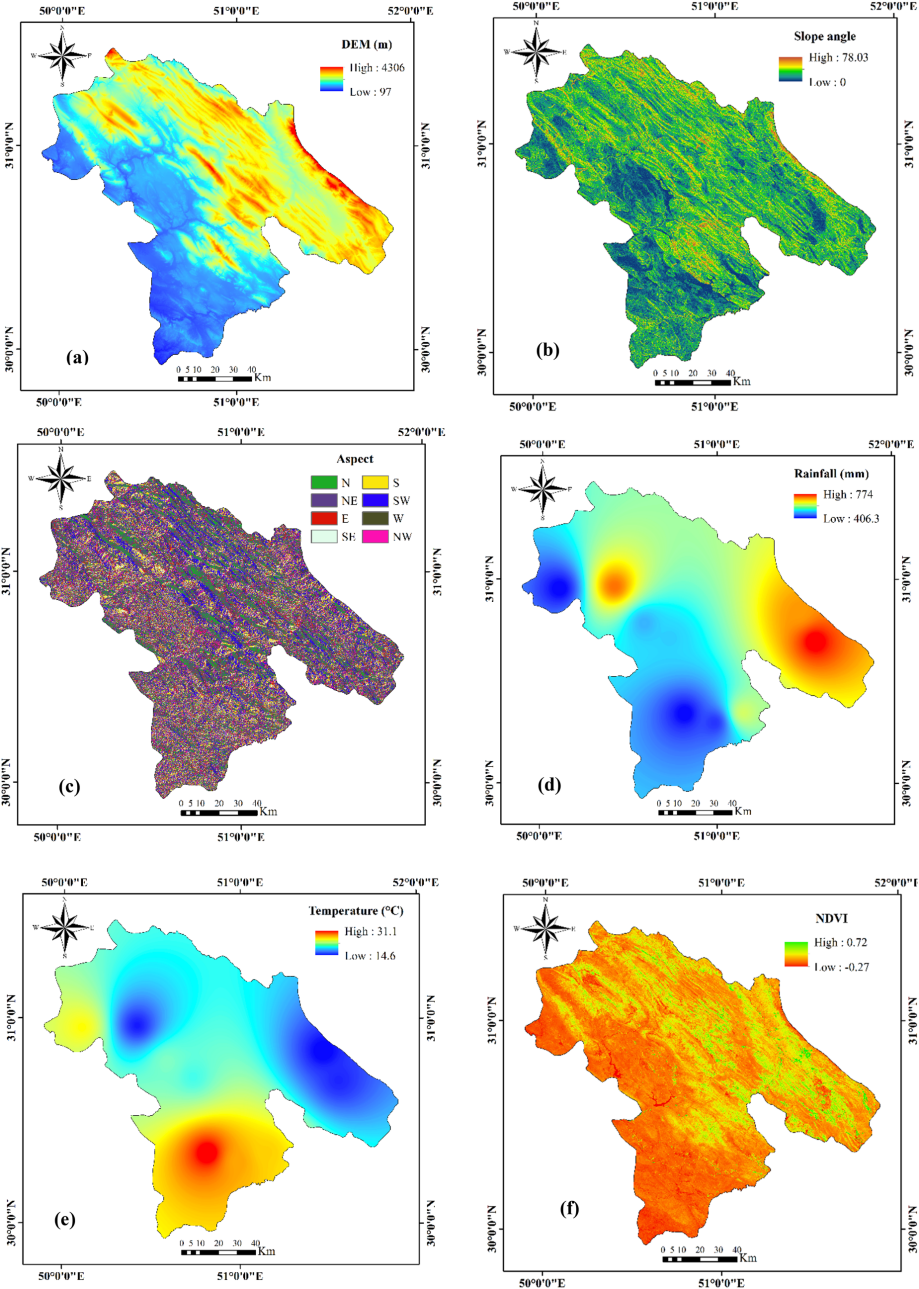

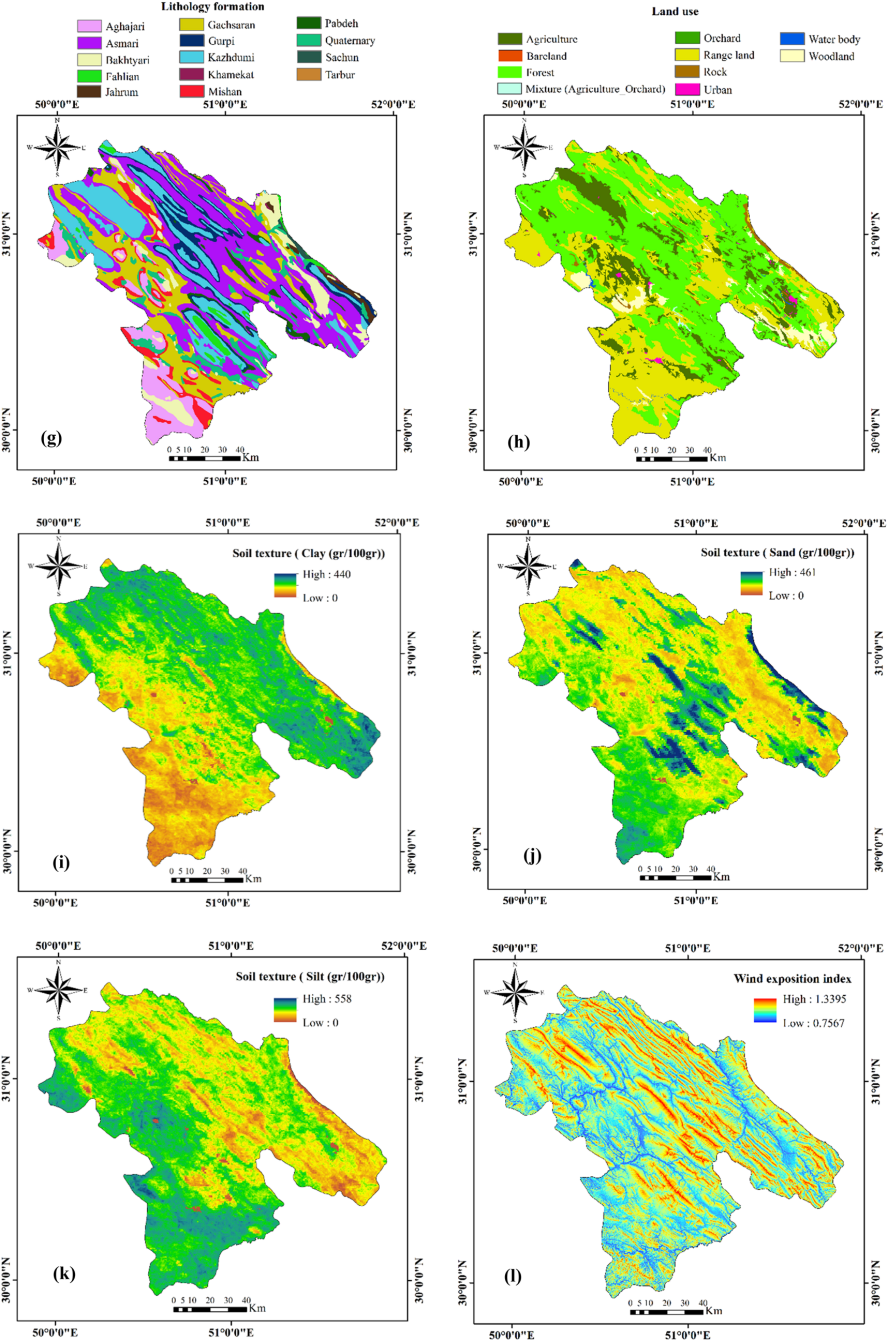

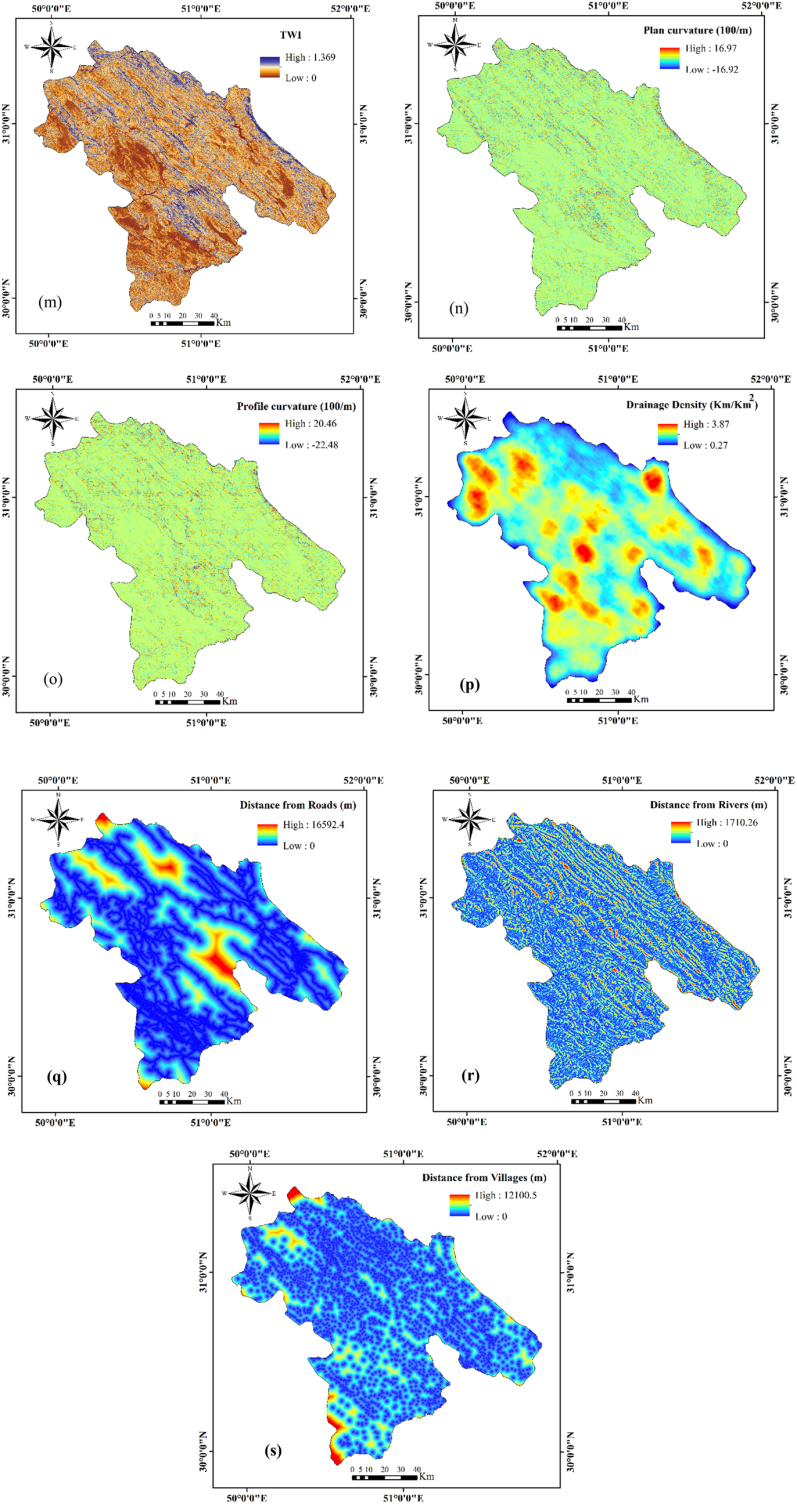
Maps of hazard factors used in this study.

Elevation is widely used as a controlling factor in natural hazard assessments^27, 67^. In this study, we used elevation factors in the preparation of flood, forest fire, and gully erosion maps. An elevation layer was prepared from ASTER satellite images (https://earthexplorer.usgs.gov/) (Fig. 9a).

#### Slope angle

The slope angle is an important contributing factor in flooding, forest fires, and gully erosion^68, 69^. In the case of forest fires, fire spreads more rapidly on steep slopes than on gentle ones^70^. In contrast, with all other things being equal, the likelihood of flooding increases with a decrease in slope, and flat and gently sloping ground in the study area is more prone to gully erosion than steep slopes^58^. A slope-angle layer was derived from DEM in ArcGIS 10.8. The slopes ranged from 0° to 78° (Fig. 9b).

#### Slope aspect

Sunlight, humidity, and temperature are affected by slope aspect; thus, aspect can be an important factor in the occurrence of floods, forest fires, and gully erosion. Eastern slopes in the study area receive sunlight earlier than western aspects, and therefore are more fire-prone^20^. The slope aspect has also been shown to be related to gully erosion through weathering mechanisms^17, 71^. We divided the aspect into nine classes: flat, north, northeast, northwest, southwest, west, east, south, and southeast (Fig. 9c).

#### Rainfall

Rainfall has been widely used as a factor in multi-hazard spatial modelling^17, 72^. Low rainfall in forested areas makes these areas susceptible to wildfire^72, 73^, whereas extreme rainfall triggers flooding^74^ and gully erosion^58^. We prepared a rainfall layer in the ArcGIS 10.8 using data for the period 2001–2020 from nine weather stations in Kohgiluyeh and Boyer-Ahmad Province (Fig. 9d). A temperature map was obtained using the IDW interpolation^75, 76^.

#### Temperature

All other things being equal, high temperatures, and a lack of rainfall rapidly dry soil, making forests in environments prone to wildfire^77^. In the study area, temperature and humidity seem to be stronger controls for forest fires in higher parts of the province than lower ones^78^. We produced an average annual temperature layer based on climate data from nine stations during the period 2001–2020 (Fig. 9e). A rainfall map was created through interpolation using the IDW method^79^.

#### Normalized difference vegetation index

The normalized difference vegetation index (NDVI) provides a measure of vegetation health^80^ and is thus an important factor in forest fire, gully erosion, and flood susceptibility assessments. We calculated the NDVI from a Sentinel-2 satellite image in Google Earth Engine using the following equation:

2 $$NDVI = \left( {NIR - RED} \right)/\left( {NIR + RED} \right)$$

where NIR and RED refer to the near-infrared and red bands, respectively (Fig. 9f).

#### Lithology

Rock permeability, which is related to lithology, may affect flood susceptibility^28, 81–83^ and gully erosion^5^. We obtained a geology map from the Geological Survey of Iran and produced a layer of 14 units in the ArcGIS 10.8 (Fig. 9g).

#### Land use

Land use changes, including deforestation and grazing, can initiate or exacerbate gully erosion^30, 84–86^, and bare lands are more susceptible to floods than naturally vegetated ones^71^. Therefore, we considered land use as a factor in the preparation of forest fire and flood maps. The land-use layer has 10 classes: agriculture, bare land, forest, mixture (agriculture and orchard), orchard, range land, rocky land, urban, water body, and woodlands (Fig. 9h). The land use map (with an accuracy of 91% prepared by the support vector machine (SVM) method) was obtained using the Landsat-8 (2019) satellite imagery.

#### Soil texture

A soil texture is considered one of the main factors in gully erosion^87^. We used data and guidelines provided by the World Soil Information Service (WoSIS, http://soilgrids.org) to prepare the soil layer in the ArcGIS 10.8. The SoilGrids map is a global digital soil mapping system that employs new machine learning techniques to map the spatial distribution of soil properties. The SoilGrids maps are available with a spatial resolution of 250 m. The SoilGrids maps are generated using more than 230,000 soil profile observations from the WoSIS database and over 400 environmental layers of Earth observation and other environmental data such as climate, land cover, and land morphology (https://www.isric.org/explore/soilgrids). The WoSIS database includes typical soil features for each soil group^88^. We defined soil texture in the study area using three percentage classes as proxies: clay, silt, and sand (Fig. 9i,j,k).

#### Wind exposition index

The wind exposition index (WEI) is a non-dimensional index used to quantify wind exposure at the land surface^89^. It takes into account the wind direction, angle of the surface earth to the horizon, and wind aspect^90^. Values larger than 1 indicate wind-exposed cells, and values less than 1 correspond to wind-shadowed cells^91^. WEI was computed from the DEM using the SAGA-GIS software (http://www.saga-gis.org/en/index.html) (Fig. 9l).

#### Topographic wetness index

The topographic wetness index (TWI) is a measure of the likelihood that surface water will move downslope^92, 93^ and is used in flood, forest fire, and gully erosion assessments. TWI was calculated from ASTER imagery using the SAGA GIS software (Fig. 9m).

#### Plan curvature

Plan curvature is the curvature of an isoline constructed from the junction of a horizontal plane and the land surface^94, 95^. This factor was considered in flood and gully erosion evaluations. A plan curvature layer was derived from the DEM using the spatial analysis extension (curvature tool) in ArcMap (Fig. 9n).

#### Profile curvature

The profile curvature is a measure of the curvature of the slope surface. It controls surface and groundwater movement, and thus is a metric for flow velocity and erosion^94, 96^. The profile curvature layer was constructed from the DEM in the ArcGIS 10.8 platform using a curvature tool (Fig. 9o). Negative and positive values indicate the concave and convex surfaces, respectively.

#### Drainage density

Drainage density (DD) is controlled by precipitation, geology, vegetation, slope, and soil, and is a factor in gully erosion and flood assessments. Drainage density scales with runoff and therefore may be an indicative factor for gully erosion^97, 98^. The drainage density layer was created from the rivers and streams in ArcGIS 10.8 using density tools (Fig. 9p).

#### Distance from roads

Distance from roads is important in flood, forest fire, and gully erosion assessments. In forest areas, the risk of fire increases with proximity to roads^21^, and gully erosion, especially in bare terrain, might be related to this variable^99^. The distance from roads was quantified using vector line distances calculated using the Euclidean distance method in ArcGIS 10.8 (Fig. 9q).

#### Distance from rivers

The probability of flooding is related to the distance from a river, especially for rivers with low storage capacity^100^. In addition, most people in the study area live near rivers; thus, there is a higher likelihood of forest fires in these areas^101^. Gully erosion in the study area is also the greatest near rivers^102^. The distance from the river layer was determined using the Euclidean distance tool in ArcGIS 10.8 (Fig. 9r).

#### Distance from villages

Distance from villages, such as distance from rivers, may be related to the probability of forest fires, as most wildfire events are human-caused events^103, 104^. Figure 9s shows the distance from the village layer produced in ArcGIS 10.8. This factor was not used in flood and gully erosion assessments.

### Multi-hazard spatial modelling

#### Boosted regression tree

The boosted regression tree (BRT) algorithm is a machine learning method based on the use of classification and regression trees in combination with a boosting algorithm^105^. Its purpose is to improve the performance of a single model by fitting and combining a large number of models for prediction^106^. It can be used to predict quantitative (regression tree) or categorized (classification tree) outcomes. In this study, the regression tree model is reinforced by assessing logical conditions, rather than a linear relationship, to predict or classify landforms. When using the BRT model, there is no need to predetermine the function forms or make statistical assumptions about the data distribution. Other advantages include the ability to make multiple predictions and determine their possible nonlinear relationships with the response variable^107^, the high speed of the model in analyzing large volumes of data, and its high capacity to analyze and classify layers. The algorithm depends on the setting options related to the reinforced trees and tree pruning. In the case of options related to reinforced trees, an important parameter is the reduction rate as a net weight for individual and reinforced trees. Optimizing the best reduction rate is also important for preventing overfitting of the predictions. Previous studies have shown that models with a reduction rate of 0.1 or less perform best^108^. The best parameters are selected based on an evaluation of their results using statistical evaluation metrics (RMSE and bias). In this study, the regression trees were programmed using R 3.5.3 statistical software (https://cran.r-project.org/bin/windows/base) with a BRT extension (see Elith et al.^109^ for details).

### Support vector machine

The support vector machine (SVM) is a supervised learning method used for classification and regression. It was proposed in 1995 based on statistical learning theory using structural risk minimization^110^. It has been widely used in recent years as it performs better than older classification methods such as perceptron neural networks^111^. The SVM classifier works by linearly classifying data; in segmenting the data, it selects the most reliable boundary^110^. The closest samples to the decision boundary, which determine the decision boundary equation, are termed support vectors^112^. The principle of structural risk minimization is applied to maximize the distance between two transient hyperplanes formed from support vectors^113^. SVM performs better on non-modeled data than the experimental risk-minimization mode, which attempts to minimize the training error. Four types of kernel functions (linear, polynomial, sigmoid, and radial basis) can be used to prepare multi-hazard maps using the SVM algorithm. We used the radial basis function in this study because of its better performance than other functions^114–116^. The SVM algorithm was programmed in the R3.5.3 statistical software with the sdm package^117^.

### Random forest

The random forest (RF) algorithm is a widely used hybrid machine learning algorithm that includes regression and classification trees^118^. The data used by the RF algorithm do not need to be changed, converted, or modified, and the algorithm controls the lost values automatically^119^. Random trees classify the input vector with each tree in the forest; the output is the class tag that receives the majority of votes. Two factors (the mean decrease in accuracy and the mean decrease in Gini) are used to determine the priority of the effect of each effective factor^120^. We implemented the RF model using the R3.5.3 statistical software with the random forest package^121^.

### Probabilistic seismic hazard analysis

Probabilistic seismic hazard analysis (PSHA) is a method used to determine the probability that a location will experience severe ground movements during an earthquake of a particular magnitude. The numerical-analytical method for PSHA was first proposed in 1968^122^ and has improved since then. Relevant seismic parameters include earthquake magnitude, return period, epicentral and source distances, and peak ground acceleration^123^. Different scales are used to quantify seismic magnitude, such as moment, surface wave, volume wave, local, and torque magnitudes. One type of PSHA, the deterministic seismic hazard analysis (DSHA), determines the maximum kinetic parameters of the ground surface in a scenario earthquake, which is described by its magnitude and source distance^124, 125^. The scenario earthquake is expected to produce the greatest ground motion, such as peak ground acceleration (PGA), at the site if it were to occur^126^. We used this method to assess built structures whose destruction would have catastrophic consequences, such as nuclear power plants and large dams. It does not provide information on the probability of a scenario earthquake, its exact location, or the duration of severe ground motions. Uncertainties in determining the magnitude and source distance of an earthquake are typically combined to calculate the probability of exceedance of a specified PGA in a certain period of time. In this study, a peak horizontal acceleration map was created for an earthquake with a 10% probability of occurrence in 50 years (the time series 1965–2015) and an average return period of 75 years^127^.

### Multi-hazard mapping

A multi-hazard map was constructed from the maps of flood hazard (FH), forest fire hazard (FFH), gully erosion hazard (GEH), and peak ground acceleration (PGA). The BRT, SVM, and RF algorithms were first used to create flood, gully erosion, and forest fire maps. Then, the results of the three algorithms were compared to select the algorithm with the best performance. Next, a PGA map was produced using PSHA. Each hazard was mapped using four groups—low, moderate, high, and very high—and subsequently reclassified into two groups: 0 and 1. Class 1 indicates high to very high hazard susceptibility, whereas class 0 indicates low and moderate susceptibility. Finally, the two-class maps for three hazards (forest fire, flood, and gully erosion) and the PGA map were integrated into a final multi-hazard map for the study area.

### Model validation

Validation of multi-hazard models is required to evaluate their accuracy and value^128^. In this study, we used the receiver operating characteristic (ROC) curve to assess the model’s predictive power. The ROC plot has two axes: the *x-axis* depicts the false-positive rate and the y-the-true-positive rate. The value of the area under the curve (AUC) ranges from 0.5 to 1.0^129, 130^, and the higher the AUC value, the greater the prediction accuracy^128, 131^. A ROC curve was created for each map using both the training and validation datasets. As mentioned previously, 70% of the hazard locations were used for model training and 30% for model validation.

### Prioritizing effective factors

We employed the mean decrease accuracy (MDA) and mean decrease Gini (MDG) to determine the priority factors for natural hazard occurrences in the study area. The variance inflation factor (VIF) and tolerance coefficient (TOL) were first computed to check for multicollinearity. Variables with values of VIF ≥ 5 and TOL < 0.1 indicate a multicollinearity problem^132^. We then determined the relative importance of these factors. According to Nicodemus^133^, the Gini index is more stable than the in-mean decrease for determining the priority of effective factors, especially in situations where there is a relationship between environmental factors. The mean decrease Gini (MDG) is defined as the sum of the Gini impurities decreasing from a specific variable normalized by trees^120^. Therefore, we also used MDG to determine the priority factors in this study.

## Data Availability

The data used in this study are available upon request to the corresponding author for reasonable use in research. All figures draw by the authors using R 3.5.3 statistical software (https://cran.r-project.org/bin/windows/base), SAGA-GIS software (http://www.saga-gis.org/en/index.html), and ArcGIS 10.8 environment (https://www.esri.com).
